# Changes of inflammation in patients with psoriatic arthritis after high intensity interval training assessed by ultrasound and MRI, a randomized controlled trial

**DOI:** 10.1186/s12891-023-06871-3

**Published:** 2023-09-19

**Authors:** Ruth Stoklund Thomsen, Tom Ivar Lund Nilsen, Glenn Haugeberg, Beathe Sitter, Arthur Kavanaugh, Susanne Juhl Pedersen, Mari Hoff

**Affiliations:** 1 Department of Neuromedicine and Movement Science, NTNU, Faculty of Medicine, Norwegian University of Science and Technology, Post Box 8905, N-7491 Trondheim, Norway; 2 Department of Circulation and Medical Imaging, NTNU, Faculty of Medicine and Health Sciences, Norwegian University of Science and Technology, Trondheim, Norway; 3 Department of Rheumatology, St Olavs Hospital, Trondheim University Hospital, Trondheim, Norway; 4 Department of Public Health and Nursing, NTNU, Norwegian University of Science and Technology, Trondheim, Norway; 5 Department of Anaesthesia and Intensive Care, St Olavs Hospital, Trondheim University Hospital, Trondheim, Norway; 6 Division of Rheumatology, Department of Internal Medicine, Hospital of Southern Norway Trust, Kristiansand, Norway; 7 Division of Rheumatology, Allergy, and Immunology, School of Medicine, University of California, San Diego, USA; 8 Copenhagen Center for Arthritis Research (COPECARE), Center for Rheumatology and Spine Disease, Rigshospitalet, Denmark

**Keywords:** Psoriatic arthritis, Exercise, Inflammation, Magnetic Resonance Imaging, Ultrasonography

## Abstract

**Background:**

In psoriatic arthritis (PsA) there is a theoretical risk of increased disease activity related to strenuous physical activity, including exercise. We evaluated the effect of high intensity interval training (HIIT) on objective measures of inflammation in PsA assessed by ultrasound (US) of peripheral joints and entheses, and by bone marrow edema (BME) on MRI of the sacroiliac joints (SIJ) and spine.

**Methods:**

We randomly assigned 67 PsA patients to an intervention group that performed structured HIIT for 11 weeks, or to a control group instructed not to change their physical exercise habits. Outcome measures included US evaluation of the total cohort and MRI in a subgroup of 41; both assessed at 3 months. We calculated the proportions with an increased US B-mode and power-doppler (PD) signal of joints and entheses and Spondyloarthritis-Research-Consortium-of-Canada (SPARCC)-BME score of the SIJ and spine for both groups.

**Results:**

Proportions with an increased US B-mode score of the joints were 32% and 28% in HIIT and control groups, respectively. Corresponding proportions of PD scores of the joints were 7% and 10% and PD scores of entheses were 32% and 31%.

The proportions with increased MRI BME of the SIJ were 6% in the HIIT group and 10% in the control group. Corresponding proportions were 6% and 5% for the MRI BME of the spine.

**Conclusion:**

In PsA patients with a low to moderate disease activity, there was no clear evidence of objectively measured increased inflammation after HIIT, as evaluated by US and MRI.

**Trial registration:**

ClinicalTrials.gov NCT02995460 (16/12/2016).

**Supplementary Information:**

The online version contains supplementary material available at 10.1186/s12891-023-06871-3.

## Background

Psoriatic arthritis (PsA) is an inflammatory chronic disease with diverse manifestations including peripheral arthritis, enthesitis, dactylitis and spondylarthritis [1]. PsA is commonly associated with obesity and the metabolic syndrome; these comorbidities can increase patients` risks of cardiovascular disease and mortality [2–5]. Physical exercise (defined as an activity that is planned, structured and repetitive, and that is aiming to improve or maintain one or more dimensions of physical fitness [6]) is recommended for patients with PsA especially due to the high prevalence of overweight/obesity, and the risk of comorbidities such as metabolic syndrome and cardiovascular disease [7, 8]. Further, physical exercise may be beneficial regarding disease activity in patients with arthritis [9]. This has primarily been demonstrated in ankylosing spondylitis [10] and rheumatoid arthritis [11]. However, in PsA there is a potential risk of increased disease activity, including worsening of enthesitis, after physical exercise due to excess impact loading and subsequent mechanical strain [12, 13]. It has been hypothesized that mechanical strain may be of etiologic relevance to enthesitis and arthritis, e.g., as an “internal Koebner phenomenon”. There are few data available on physical exercise in patients with PsA. However, beneficial effect has been demonstrated regarding functional capacity, general well-being, fatigue, and quality of life [14, 15]. In addition, it seems that physical exercise is well tolerated measured by disease activity markers and patient reported outcomes [14]. However, no studies have demonstrated a stable disease activity in PsA assessed by objective measurements after physical exercise. Knowledge about the effects on inflammatory activity after physical exercise in PsA is crucial to implementing optimal and appropriate recommendations.

Traditional physical examination has limitations regarding the identification of the source of musculoskeletal signs and symptoms in PsA. This relates in some measure to difficulties differentiating inflammation driven signs and symptoms from those that are non-inflammatory or related to damage [16]. Thus, evaluating the burden of enthesitis, peripheral arthritis and inflammatory back pain is challenging [17]. Ultrasonography (US) is a more sensitive tool than clinical examination for visualization of inflammatory changes in peripheral joints and entheses in patients with PsA [16]. Further, swollen joints but not tender joints are associated with arthritis assessed by US [18]. In PsA, joint tenderness has a low association with imaging signs of inflammation, particularly in patients with high self-reported pain [19].

Axial inflammation in spondyloarthritis (SpA) and PsA is traditionally evaluated by the composite scores BASDAI and ASDAS [20]. However, they are likely to be affected by peripheral disease activity as well [21]. Magnetic resonance imaging (MRI) provides the most sensitive method for detection of axial inflammation [22]. Both MRI and US have been validated as tools reliable of demonstrating inflammatory activity in PsA [23–25].

The aim of this study was to evaluate the impact of high intensity interval training (HIIT) on objective measures of inflammation in PsA assessed by US of peripheral joints and entheses as well as bone marrow edema (BME) in MRI of the sacroiliac joints (SIJ) and spine.

## Methods

### Design

Results are based on analyzes of data from a study which previously has been described in detail. In short, this was a randomized controlled trial (RCT) with two parallel groups, comparing an intervention group performing HIIT three times per week for 11 weeks with a control group with no change in pre-study physical exercise habits. Randomization of the total study sample was done in three consecutive blocks. Sample size was calculated according to the primary outcome in the original study [15]. The study was conducted in compliance with the Helsinki Declaration and all the patients provided a written informed consent. The trial was approved by the regional ethics committee, REK south-east, Norway (RECnr 2012/1646).

Trial registration: NCT02995460 (16/12/2016).

### Patients

Eligible patients were between ages 18–65 years and fulfilled the ClASsification for Psoriatic ARthritis (CASPAR) criteria. The exclusion criteria included: patients with inability to exercise; patients with unstable ischemic cardiovascular disease or severe pulmonary disease; an anticipated need for a change in synthetic or biologic Disease Modifying Anti Rheumatic Drugs (DMARDs) during the intervention period (a change in corticosteroid doses and intra-articular corticosteroid injections were allowed until four weeks before follow up); pregnancy, breastfeeding; and drug or alcohol addictions [15]. All the participants provided a written informed consent.

### Intervention

The exercise intervention was performed as a supervised HIIT program starting with 10 min of warm up, followed by four times four minutes exercise at 85–95% of HR_max_ interrupted by three minutes exercise at 70% of the HR_max_ [26]. The supervised HIIT was performed on a stationary bicycle twice a week with an intermitting day of rest. Additionally, the participants did one self-guided HIIT a week [15].

### Ultrasonography protocol

US evaluations were performed by a rheumatologist experienced in US (RST) at baseline and at 3 months follow up. The duration of each US examination was approximately 30–45 min. The sonographer was blinded with respect to group allocation at baseline but not at follow up and was aware of the clinical results at both timepoints. Brightness mode (B-mode) and Power Doppler (PD) sonography were performed at 34 mandatory joints (bilateral metacarpo-phalangeal 1–5, radio-carpal, inter-carpal, radio-ulnar, knees, talo-crural, subtalar, talo-navicular, metatarso-phalangeal 1–5) with the Norwegian ultrasonographic atlas [27] as reference and additional joints found to be swollen or tender by 66/68 joint count. Further, 10 mandatory entheses (bilateral quadriceps, proximal and distal patellar tendons, Achilles, plantar fasciae) and additionally entheses found to be tender by clinical examination of 19 other entheses (first and seventh costosternal joints, anterior superior iliac spine, iliac crest, fifth lumbar spinous process, posterior superior iliac spine, lateral and medial epicondyle, triceps, great trochanter) were scanned for PD activity [28]. B-mode and PD signals in joints and entheses were semi quantitatively graded 0–3 [27]. The US examinations were performed using a GE Logic E9 ultrasound device with two multifrequency linear transducers (4–15 MHz and 2–8 MHz).

### Magnetic resonance imaging

A subset of the total study PsA cohort, including patients from the second and third randomization block, underwent MRI of the SIJ and spine at baseline, and at 3 months of follow up, at average 13 (range: 8 – 26) days after completion of the intervention. MRIs were obtained using two 1.5 T routine whole body MR scanners. A Short Tau Inversion Recovery (STIR) sequence and a T1-weighted turbo spin-echo sequence (TSE) based on a standardized protocol were applied in the semi-coronal scan plane of the SIJs and in the sagittal scan plane for the spine were used for examination of BME [29, 30].

The data on MRI spine has previously been methodologically described [31].

### SPARCC scoring

A rheumatologist (RST) trained in the Spondyloarthritis Research Consortium of Canada (SPARCC) scoring methods and blinded to group allocation, timepoints and clinical outcomes, scored the STIR images of the SIJ and the spine according to the SPARCC SIJ and Spine MRI Indices for inflammation [29, 30]. In the SIJ, the six consecutive slices covering the cartilaginous part of the joints, were scored. The total maximum SPARCC score is 72 for all six slices of SIJ. For the spine, the six most abnormal disco-vertebral levels on the STIR sequence were selected. Three consecutive sagittal slices, that represent the most abnormal slices for each level, were chosen for scoring at that level. The total maximum SPARCC score is 108 for all six levels of the spine. A score less than 2 is defined as being in remission for both SIJ and Spine [32]. The minimal important change is 2,5 and 5 in SPARCC BME score for SIJ and spine, respectively [32, 33].

### Statistics

Descriptive statistics are presented as mean ± SD or as median and interquartile range for non-normally distributed variables.

The difference in scores between baseline and follow up were used to classify patients as having a worse or unchanged/improved outcome in US and SPARCC BME scores. For US, a worsening was defined as an increase of one unit in B-mode or PD scores. A worsening was defined by SPARCC BME scores if scores had positive values of the minimal important change criterion or higher. The proportions with an increased US and SPARCC BME scores were calculated in percent with 95% confidence intervals (CI) for each group. Further, logistic regression analysis was performed to calculate the odds ratios (ORs) with 95% CIs for worsening in the US and SPARCC BME scores. To evaluate the actual difference in scores, the mean change between baseline and follow up was calculated for all scores in each group. Further, linear regression was performed to evaluate whether there was a difference in mean change between the groups.

All statistical analyzes were conducted using StataMP 16 (16.1 Copyright 1985–2019 StataCorp LLC, StataCorp, 4905 Lakeway Drive, College Station, Texas 77,845 USA).

## Results

For the US analyzes, 67 patients with PsA were included (32 in the intervention and 35 in the control groups). Recruitment, randomization and exclusions in the main study were as previously described [15]. Of the 67 patients, 10 patients dropped out before the follow up, leaving 57 patients (28 in the intervention and 29 in the control groups) for evaluation of changes in US scores. A subgroup of 41 patients (21 in the intervention and 20 in the control groups) agreed to undergo MRI of the SIJs and the spine at baseline. Four of those dropped out of the study before the MRI could be done at follow up, leaving 37 patients (17 in the intervention and 20 in the control groups) for evaluation of changes in BME scores. Overall, the included patients had a low to moderate disease activity. Baseline characteristics of the patients are presented in Table 1.

**Table 1:** Baseline characteristics in the intervention and control groups of patients with psoriatic arthritis

	Total sample		MRI subgroup	
	Intervention (*N* = 32)	Control (*N* = 35)	Intervention (*N* = 21)	Control (*N* = 20)
Age, years Mean (SD)	50.7 (11.0)	45.6 (11.5)	51.6 (9.1)	44.4 (12.9)
Female n (%)	21 (66)	22 (63)	17 (81)	12 (60)
Disease duration, years Median (IQR)	5.5 (2–12)	3 (2–11)	5 (2–11)	3.5 (1–12.5)
HLA-B27 positive n (%)	4 (13)	4 (13)	4 (19)	3 (15)
Synthetic DMARDS n (%)	29 (91)	28 (80)	21 (100)	16 (80)
Biologic DMARDS n (%)	11 (34)	10 (29)	6 (29)	6 (30)
BMI (kg/m^2^) Mean (SD)	28.6 (4.1)	28.0 (4.5)	29.4 (4.1)	26.9 (4.3)
HS-CRP (mg/L) Median (IQR)	1.67 (0.9–4.5)	1.87 (0.9–4.7)	1.8 (0.8–4.5)	2.2 (0.8–4.1)
**PGA** VAS 0–100 mm Mean (SD)	37.4 (23.4)	42.9 (20.8)	43.6 (24.2)	42.4 (20.7)
DAPSA68 categories, n (%)				
1 remission	5 (16)	4 (11)	1 (5)	2 (10)
2 low	11 (34)	9 (26)	7 (33)	5 (25)
3 moderate	11 (34)	20 (63)	9 (43)	12 (60)
4 high	5 (16)	2 (6)	4 (19)	1 (5)
Tender joints 66 Median (IQR)	4.5 (1–9)	6 (1–9)	7 (1–10)	6 (1–9.5)
Swollen joints 68 Median (IQR)	0 (0–1)	0 (0–2)	0 (0–1)	1 (0–2.5)
Pain VAS 0–100 mm Mean (SD)	35.3 (21.0)	39.2 (22.8)	40.5 (21.6)	36.0 (21.2)
ASDAS-CRP Mean (SD)	2.08 (0.96)	2.18 (0.89)	2.3 (1.0)	2.1 (0.9)
SPARCC-EI Median (IQR)	3 (1–6)	3 (0–5)	4 (1–6)	2.5 (0.5–3.5)
MHAQ Median (IQR)	0.32 (0- 0.69)	0.38 (0.25- 0.63)	0.38 (0–0.88)	0.38 (0.25–0.63)
US joint BM Median (IQR)	2 (0–7.5)	3 (1–7)	2 (0–8)	2.5 (0–8.5)
US joint PD Median (IQR)	0 (0–1.5)	0 (0–2)	0 (0–2)	0 (0–2.5)
US entheses PD Median (IQR)	0 (0–1)	0 (0–1)	0 (0–1)	0 (0–1)
SIJ-BME-SPARCC Median (IQR)			0 (0–0)	0 (0–1.5)
SIJ-BME-SPARCC n				
< 2			16	15
≥ 2			5	5
Spine-BME-SPARCC median (IQR)			4 (0–11)	4 (0–6)
Spine-BME-SPARCC n				
< 2			8	7
≤ 2 – 10			7	9
> 10			6	4

*MRI* magnetic resonance imaging, *SD* standard deviation, *IQR* interquartile range, *DMARDs* disease-modifying antirheumatic drugs, *BMI* Body Mass Index, *Hs-CRP* high-sensitivity C-reactive protein, *PGA* patient`s global assessment, *VAS* visual analogue scale, *DAPSA68* Disease Activity index for Psoriatic Arthritis of 66/68 joints, DAPSA68 categories: 1. Remission (0–4) 2. Low (5–14) 3. Moderate (15–28) 4. High (> 28), *ASDAS-CRP* Ankylosing Spondylitis Disease Activity Score using the CRP level., *SPARCC-EI* Spondyloarthritis Research Consortium of Canada enthesitis index (0–16), *US joint BM* ultrasound of joints, brightness-mode, *US joint PD* ultrasound of joints, Power Doppler, *US entheses PD* ultrasound of entheses, Power Doppler, *SIJ-BME-SPARCC* SPARCC index of sacroiliac joint bone marrow edema (0–72, remission < 2), *Spine-BME- SPARCC* SPARCC index of spine bone marrow edema (0–108, remission < 2)

Proportions with an increased B-mode score of the joints were 32% (95% CI, 15 to 49) in the HIIT group and 28% (95% CI, 11 to 44) in the control group. For the PD scores of the joints the proportions were 7% (95% CI, -2 to 17) and 10% (95% CI, -1 to 21) in HIIT and control groups, respectively. Further, the PD scores of entheses increased in 32% (95% CI, 15 to 49) of cases in the HIIT group and 31% (95% CI, 14 to 48) in the control group (Table 2).

**Table 2:** Proportions with increased B-mode and power doppler scores of joints and power doppler scores of entheses, and Odds ratio (OR) for a worsening comparing the intervention and control groups with psoriatic arthritis after the high intensity interval training

	Increased score Cases/total	Increased score Cases % (95% CI)	Risk of worsening Intervention compared to control groups OR (95% CI)
Intervention *n* = 28^*^			
US BM joint	9/28	32% (15–49)	1.24 (0.40–3.88)
US PD joint	2/28	7% (-2–17)	0.67 (0.10–4.33)
US entheses PD	9/28	32% (15–49)	1.05 (0.34–3.22)
Control *n* = 29^*^			
US BM joint	8/29	28% (11–44)	
US PD joint	3/29	10% (-1–21)	
US entheses PD	9/29	31% (14–48)	

*US* ultrasound, *BM* brightness-mode, *PD* power doppler, *CI* confidence interval

^*^Missing at follow up are not included (4 in intervention and 6 in control groups)

The proportions with increased MRI BME of the SIJ were 6% (95% CI, -5 to 17) in the HIIT group and 10% (95% CI, -3 to 23) in the control group. For the MRI BME of the spine the proportions of increased scores were 6% (95% CI, -5 to 17) and 5% (95% CI, -5 to 15) in HIIT and control groups, respectively (Table 3).

**Table 3:** Proportions with increased bone marrow edema in MRI of the sacroiliac joints and spine, and Odds ratio (OR) for a worsening comparing the intervention and control subgroups with psoriatic arthritis after the high intensity interval training

	Increased score Cases/total	Increased score Cases % (95% CI)	Risk of worsening Intervention compared to control groups OR (95% CI)
Intervention *n* = 17			
SIJ	1/17	6% (-5–17)	0.56 (0.05–6.51)
Spine	1/17	6% (-5–17)	1.19 (0.07–20.54)
Control * n* = 20			
SIJ	2/20	10% (-3–23)	
Spine	1/20	5% (-5–15)	

*SIJ* sacroiliac joint, *CI* confidence interval

**Fig. 1:**
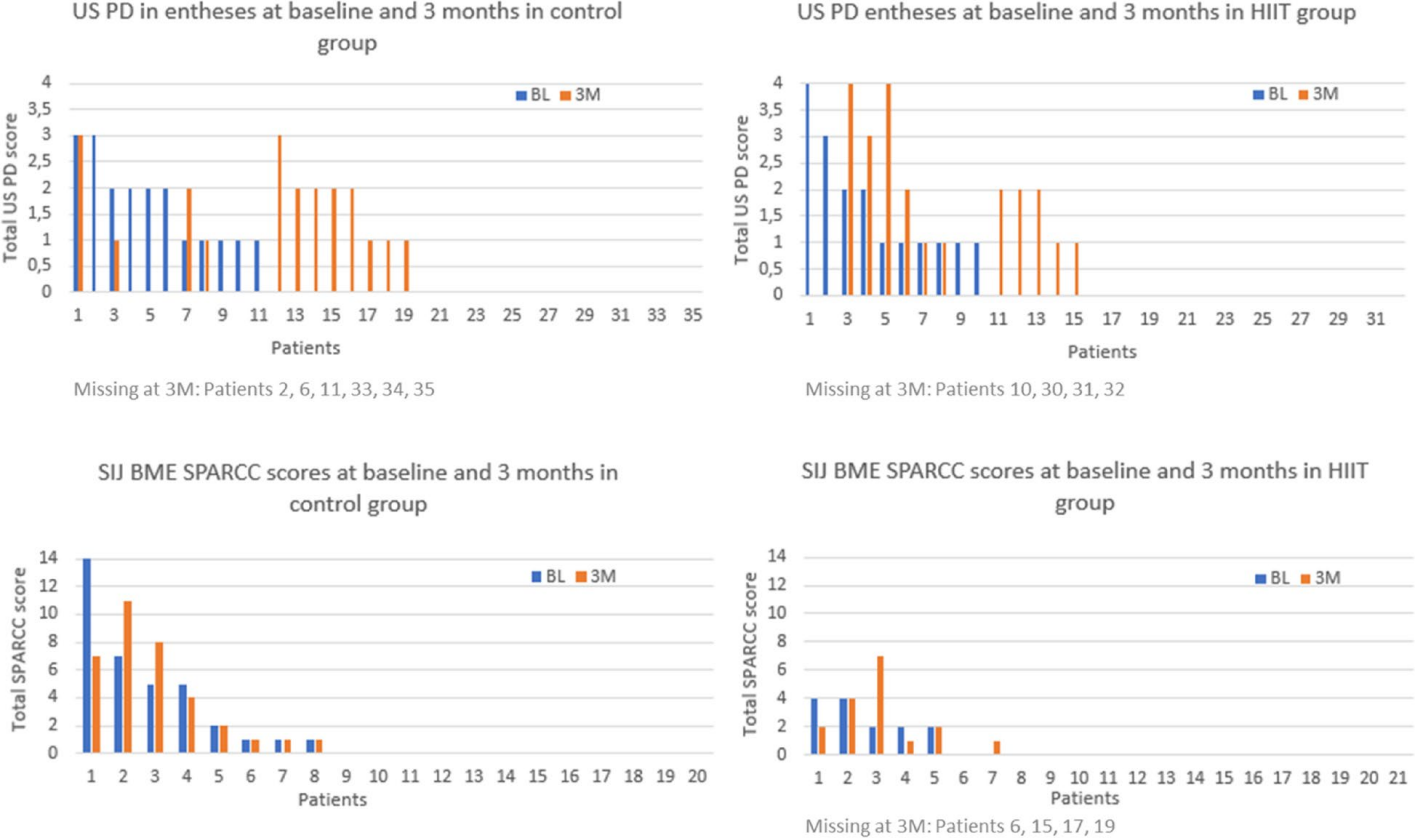
Changes in US power doppler scores of the entheses and MRI-BME-SPARCC scores of the SIJ for each individual in the control and HIIT groups at baseline and 3 months. Each number at the x-axis represents individuals in a descending order according to the baseline value for each variable. In the case of a missing column, it either represents the value 0 or missing for that individual. When representing a missing value, it is noted underneath each chart. US – ultrasound, PD – power doppler, SIJ – sacroiliac joints, BME—bone marrow edema, SPARCC—Spondyloarthritis-Research-Consortium-of-Canada, HIIT—high intensity interval training, BL- baseline, 3M—3 months

The changes for each individual in both groups from baseline to three months in PD of entheses and MRI BME of SIJ are illustrated in Fig. 1. In the Supplementary files, figures S 1 and S 2 are illustrations of the corresponding changes in US B-mode and PD of the joints as well as MRI BME of the spine.

The OR for a worsening of disease activity measured by US in the B-mode and PD scores of the joints was 1.24 (95% CI, 0.40 to 3.88) and 0.67 (95% CI, 0.10 to 4.33), respectively, when comparing the HIIT group with the control group (Table 2). The OR for a worsening in PD score of entheses was 1.05 (95% CI, 0.34 to 3.22) comparing the HIIT group to the controls.

The OR for worsening in MRI assessed BME was 0.56 (95% CI, 0.05 to 6.51) for the SIJs and 1.19 (95% CI, 0.07 to 20.54) for the spine, comparing the HIIT group to controls (Table 3).

The differences in mean changes of the scores were -0.18 (95% CI, -2.61 to 2.25) for US B-mode of joints; 0.53 (95% CI, -1.23 to 2.29) for US PD of joints; -0.10 (95% CI, -0.80 to 0.60) for US PD of entheses; 0.23 (95% CI, -0.94 to 1.39) for BME in SIJ; 3.51 (95% CI, -2.83 to 9.85) for BME in the spine; comparing the HIIT group with the control group.

## Discussion

In this RCT, there was no clear evidence of an increased risk of inflammation after three months of HIIT, evaluated by objective measures such as US of the peripheral joints and entheses, as well as by MRI BME of the SIJ and spine, comparing the HIIT group with controls.

It is crucial to implementing recommendations on physical activity and exercise to be aware of potentially harmful effects of this activity on inflammation in PsA. It has been hypothesized that mechanical stress in an inflammatory environment can promote the onset of enthesitis [34]. Animal models mimicking human PsA, suggests deleterious effects of biomechanical stress on Achille’s tendon insertion generating local inflammation and an excess of bone formation [12]. Results from similar studies in patients with PsA are contradictory. One study suggests an association between physical exercise and structural damage to the Achilles tendon but did not show associated inflammation [35]. Further, this study found no association between clinical enthesitis and achilles enthesitis defined by US in patients with PsA. In another study, PsA patients reporting regular physical activity did not have an increased risk of enthesitis evaluated by US. However, the patients who reported avoidance of any physical activity had less enthesis inflammation assessed by US [36].

In healthy individuals, both at low activity and after physical activity, US findings of active inflammation at the lower limb entheses have been observed [37–39]. However, one study found that patients with SpA have a significantly higher degree of enthesitis evaluated by US compared to active athletes [40]. This could be explained by a greater ability to reverse inflammation induced by mechanical stress in healthy individuals [41]. Thus, it seems reasonable to propose a systematic rest before US evaluation of enthesis after physical activity [40].

Reducing the inflammatory environment with treatments could reduce the risk of enthesitis. Thus, it seems rational for patients to be in remission before starting any vigorous physical activity. Properly adapted and supervised exercise could also minimize this risk [14].

In our study, we did not evaluate for structural changes. However, the proportions with an increase of inflammation in the entheses were equal in both groups. This could be due to a low to minimal disease activity at baseline due to good medical treatment, thus protecting the patients from a flare after the HIIT. Also, the HIIT exercise was performed on a stationary bicycle that minimizes the mechanical stress to lower limbs and back. Finally, the timing of the US evaluation after the HIIT could be of importance as it was performed 1–2 weeks after the last HIIT.

Only a few studies have evaluated the impact of physical activity on joint inflammation in PsA [14, 42]. In our main study we observed a stable DAS44 and pain score after HIIT assuming a non-deleterious impact on joint inflammation [15]. These findings are supported by the US results in our present study although the proportion with an increase in US B-mode was slightly higher in the HIIT group. However, the proportion with an increase in US PD was lower in the HIIT group compared to controls.

MRI BME in spine and SIJ are not unique findings in PsA and SpA. MRI BME in the SIJ is frequently seen in other conditions as well, such as in patients with nonspecific back pain and in healthy subjects such as athletes and military recruits. And especially in postpartum women with buttock/pelvic pain a positive MRI BME in SIJ has been observed [43, 44]. Mechanical strain can provoke BME in SIJ in healthy individuals. However, in a large cross-sectional study self-reported physical activity of more than 2 h per week did not increase MRI BME of neither SIJ nor spine [44]. Still, it was found that a high BMI is associated with an increased risk of BME. In SpA and PsA, clinical studies indicate a beneficial effect of physical exercise on axial inflammation evaluated by BASDAI and ASDAS-CRP [42, 45].

Our patients with PsA had a low degree of BME in both the SIJ and spine at baseline and they had a moderate axial inflammation measured by ASDAS-CRP. Further, only 15% were HLA B27 positive. The axial phenotype of PsA is associated with HLA B27 [46]. Still, one study found that only 25%-30% with axial involvement in PsA are HLA B27 positive [47]. However, the lower proportion of HLA B27 positive PsA patients in our study may indicate that only a minority of the patients had an axial phenotype of PsA and thus explain the stable degree of BME after HIIT. We observed a smaller proportion with an increase in MRI BME of the SIJ in the HIIT group compared to controls.

A strength of this study was the randomized and longitudinal design. Further, the use of objective evaluation of inflammation by US and MRI covering both the peripheral joints, entheses and axial skeleton, is reflecting the whole specter of potential regions with inflammation in PsA. Also, the MRI reader was blinded to group allocation, timepoint and clinical outcomes. The chosen mode of intervention, the HIIT, is a standardized method of physical exercise and may be a more reliable measure of physical exercise compared to self-report of activity.

Adherence is a major barrier to interventions such as physical activity. However, the dropout rate was only 6–7% in both groups making the risk estimates more reliable. We have no information about the activity of the control group. However, the VO2 max (an objective measure of cardiorespiratory fitness) capacity increased significantly in the HIIT group compared to controls, indicating that the HIIT group did more physical exercise compared to controls, data not shown [48].

Other limitations to this study imply the small sample size, especially regarding the subset of participants with MRI in which only two thirds of the study cohort are included. Accordingly, the CIs are wide, which may imply a lack of power to observe small changes and making the proportions and risk estimates imprecise.

Further, the sonographer was not blinded to group allocation and clinical scores at follow up and US findings were not confirmed by another sonographer. However, previous studies in US found a good interrater reliability among skilled sonographers [49] and using a standardized protocol as in this study, facilitates the accuracy [28].

The external validity may be questioned as patients volunteering to this kind of activity may not previously have experienced axial or entheses inflammation to a higher degree, as that might have prevented them from attending.

## Conclusion

In PsA patients with a low to moderate disease activity, there was no clear evidence of an increased risk of objectively measured inflammation evaluated by US and MRI after HIIT, comparing the intervention group to controls. This supports that HIIT is safe in PsA without increasing disease activity, at least in patients with a low to moderate disease activity.

### Supplementary Information

**Additional file 1:** **Figure S1.** Changes in US B-mode and power doppler scores of the joints for each individual in the control and HIIT groups at baseline and 3 months.

**Additional file 2:** **Figure S2.** Changes in MRI BME SPARCC scores of the spine for each individual in control and HIIT groups at baseline and 3 months.

## Data Availability

The data from the current study can be made available upon request to the corresponding author and after approved application to the ethics committee.

## Abbreviations

PsA: Psoriatic Arthritis
HIIT: High Intensity Interval Training
BME: Bone Marrow Edema
US: Ultrasonography
SIJ: Sacroiliac Joint
PD: Power Doppler
SPARCC: Spondyloarthritis-Research-Consortium-of-Canada
MRI: Magnetic Resonance Imaging
SpA: Spondyloarthritis
BASDAI: Bath Ankylosing Spondylitis Disease Activity Index
ASDAS: Ankylosing Spondylitis Disease Activity Score
RCT: Randomized Controlled Trial
CASPAR: ClASsification for Psoriatic ARthritis
DMARDs: Disease Modifying Anti Rheumatic Drugs
HRmax: Heartrate maximum
B-mode: Brightness mode
STIR: Short Tau Inversion Recovery
TSE: Turbo spin-echo
OR: Odds ratio
VO2max: Maximal oxygen uptake
