# Supplementary material for: Changes of inflammation in patients with psoriatic arthritis after high intensity interval training assessed by ultrasound and MRI, a randomized controlled trial
Source: BMC Musculoskelet Disord. 2023 Sep 19;24:743. doi: 10.1186/s12891-023-06871-3 (PMC10508016; doi:10.1186/s12891-023-06871-3)

## Slide 1
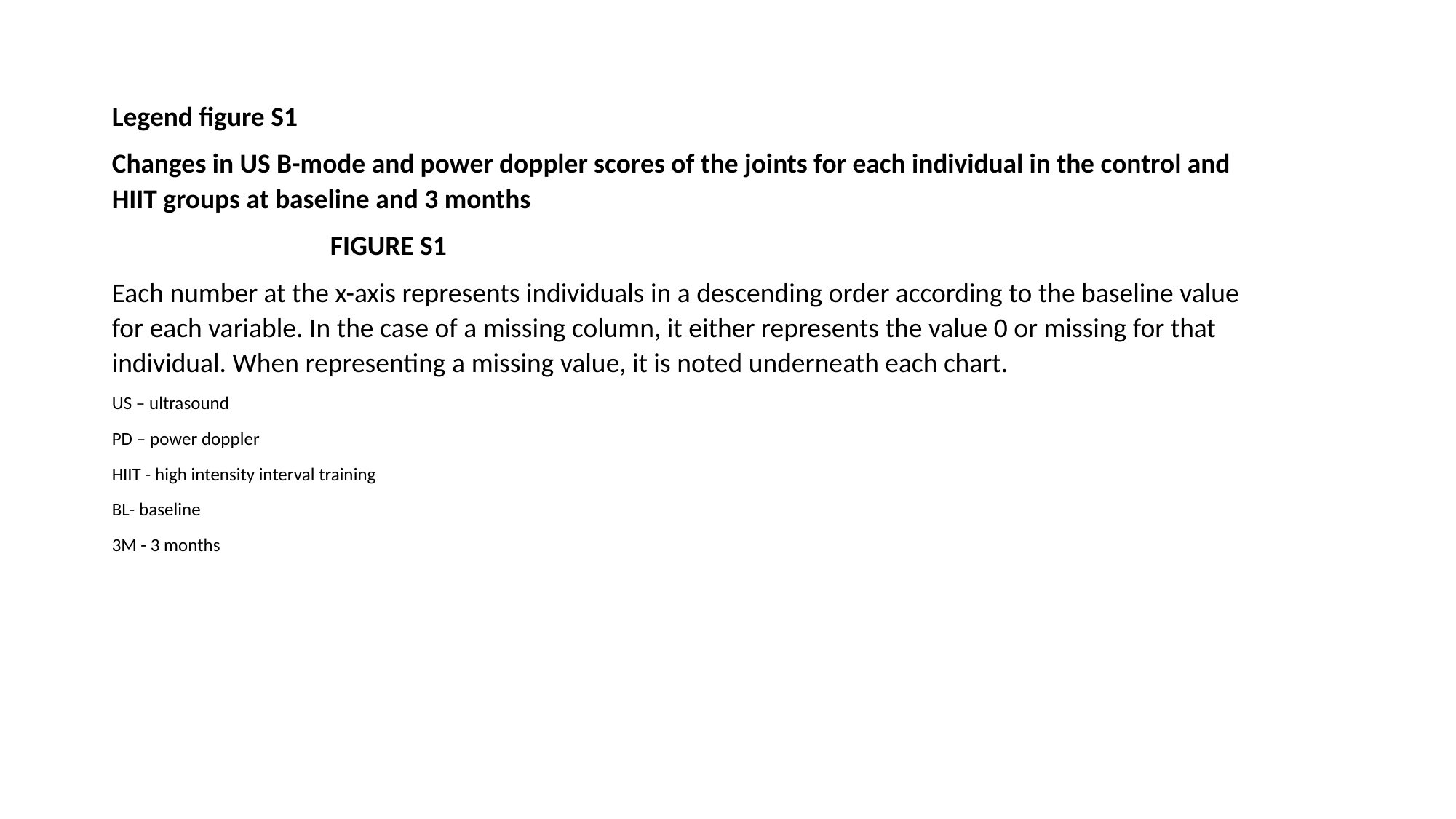

Legend figure S1
Changes in US B-mode and power doppler scores of the joints for each individual in the control and HIIT groups at baseline and 3 months
		FIGURE S1
Each number at the x-axis represents individuals in a descending order according to the baseline value for each variable. In the case of a missing column, it either represents the value 0 or missing for that individual. When representing a missing value, it is noted underneath each chart.
US – ultrasound
PD – power doppler
HIIT - high intensity interval training
BL- baseline
3M - 3 months

## Slide 2
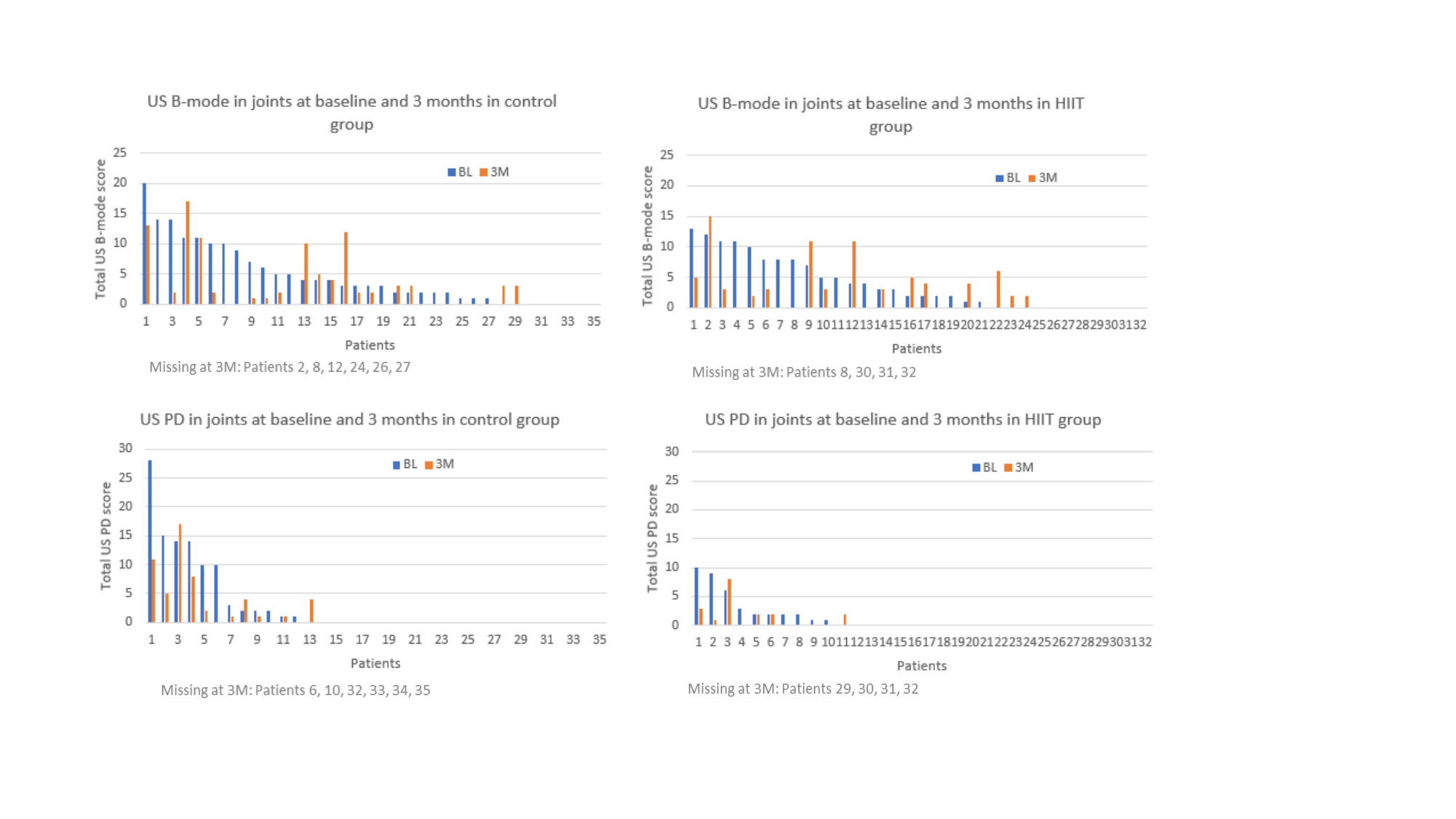

Supplement: Supplementary file 1 — Additional file 1: Figure S1. Changes in US B-mode and power doppler scores of the joints for each individual in the control and HIIT groups at baseline and 3 months. [file 12891_2023_6871_MOESM1_ESM.pptx]
